# Nonsynonymous Mutations in Intellectual Disability and Autism Spectrum Disorder Gene PTCHD1 Disrupt *N*-Glycosylation and Reduce Protein Stability

**DOI:** 10.3390/cells13020199

**Published:** 2024-01-21

**Authors:** Connie T. Y. Xie, Stephen F. Pastore, John B. Vincent, Paul W. Frankland, Paul A. Hamel

**Affiliations:** 1 Department of Laboratory Medicine & Pathobiology, University of Toronto, Toronto, ON M5S 1A8, Canada; 2 Molecular Neuropsychiatry & Development (MiND) Lab, Campbell Family Mental Health Research Institute, Centre for Addiction and Mental Health, Toronto, ON M5T 1RS, Canada; 3 Institute of Medical Science, University of Toronto, Toronto, ON M5S 1A8, Canada; 4 Department of Psychiatry, University of Toronto, Toronto, ON M5T 1R8, Canada; 5 Program in Neurosciences and Mental Health, The Hospital for Sick Children, Toronto, ON M5G 1X8, Canada; 6 Department of Psychology, University of Toronto, Toronto, ON M5S 3G3, Canada; 7 Department of Physiology, University of Toronto, Toronto, ON M5S 1A8, Canada

**Keywords:** PTCHD1, autism spectrum disorder, mutations, post-translational processing, protein stability, neurons

## Abstract

*PTCHD1* has been implicated in Autism Spectrum Disorders (ASDs) and/or intellectual disability, where copy-number-variant losses or loss-of-function coding mutations segregate with disease in an X-linked recessive fashion. Missense variants of *PTCHD1* have also been reported in patients. However, the significance of these mutations remains undetermined since the activities, subcellular localization, and regulation of the PTCHD1 protein are currently unknown. This paucity of data concerning PTCHD1 prevents the effective evaluation of sequence variants identified during diagnostic screening. Here, we characterize PTCHD1 protein binding partners, extending previously reported interactions with postsynaptic scaffolding protein, SAP102. Six rare missense variants of PTCHD1 were also identified from patients with neurodevelopmental disorders. After modelling these variants on a hypothetical three-dimensional structure of PTCHD1, based on the solved structure of NPC1, PTCHD1 variants harboring these mutations were assessed for protein stability, post-translational processing, and protein trafficking. We show here that the wild-type PTCHD1 post-translational modification includes complex *N*-glycosylation and that specific mutant proteins disrupt normal *N*-link glycosylation processing. However, regardless of their processing, these mutants still localized to PSD95-containing dendritic processes and remained competent for complexing SAP102.

## 1. Introduction

High-throughput and increasingly precise genomic approaches have identified myriad genetic loci involved in Autism Spectrum Disorder (ASD) [1,2]. The biological pleiotropy of these defined loci, using cytogenetics, linkage analysis, whole-genome linkage, or association, as well as whole-genome or exome sequencing, underlines the complexity of ASD [3]. The ASD-associated gene at Xp22.11, *PTCHD1*, was identified by several groups [4,5,6,7,8] using distinct approaches including one that indicated that *PTCHD1*-related mutations may occur in approximately 1% of individuals with ASD [6].

*PTCHD1* encodes an 888 amino acid protein that is structurally similar to the class of the resistance–nodulation–cell division (RND) superfamily of transporters (for a review, see [9]) as well as two cholesterol transporters related to Niemann–Pick syndrome type C protein, NPC1 [10,11,12]. While related to the receptors of the Hedgehog (Hh)-ligands, Ptch1 and Ptch2 [13,14,15], we and others have not yet been able to show that PTCHD1 plays a regulatory role in the Hh-pathway [16,17] and there is a current lack of evidence showing that PTCHD1 directly binds to or facilitates cholesterol fluxes. Regardless, PTCHD1 encodes a protein predicted to harbor two “Ptch1-domains”. These modules, that are juxtaposed in the membrane, are defined by five transmembrane α-helices flanked by luminal and cytoplasmic regions. The luminal domains exhibit sequence similarities to the analogous regions in NPC1 [11,18] and Ptch1 [19,20,21] that suggest they may have highly similar three-dimensional structures. In a multiple-sequence alignment, PTCHD1 showed 21.17% similarity in amino acid sequence identity with NPC1 and 21.65% with PTCH1 (Clustal Omega, www.ebi.ac.uk, accessed on 3 October 2023). However, like all other members of this class of transmembrane proteins, the cytoplasmic regions of PTCHD1 are unrelated to those in, for example, any of the Ptch-proteins or NPC1 [13]. In the case of PTCHD1, the last four amino acids at its C-terminus encode a unique motif predicted to bind PDZ-domain-containing factors [17]. Using a yeast two-hybrid screen, this motif was used previously to isolate PSD95 (*DLG4*) and SAP102 (*DLG3*), proteins localized to dendritic spines in the post-synaptic region where a large number of factors involved in synaptic transmission are organized [17]. Indeed, a GST-fusion protein encoding the C-terminus of PTCHD1 binds PSD95, although the localization of PTCHD1 to dendritic spines did not appear to be dependent on the PDZ-binding region in its C-terminus, consistent with PTCHD1 transport to dendritic spines being mediated by distinct mechanisms and regions of the protein.

We report here the identification and characterization of a series of point mutations in PTCHD1 derived from patients with ASD or other neurodevelopmental disorders. We further show that for a number of mutations in PTCHD1, these align with sequences crucial for the complexing with cholesterol in the structurally related protein Ptch1. Despite these specific mutations altering the processing of the newly synthesized proteins as well as the protein stability of PTCHD1, they do not produce defects to its ability to localize to structures containing PSD95.

## 2. Materials and Methods

### 2.1. Cell Culture

Human embryonic kidney 293T (HEK-293T) cells (a kind gift from Prof. S. Girardin, University of Toronto) were cultured in Dulbecco’s Modified Eagle Medium with 10% fetal bovine serum (FBS) (Wisent, Saint-Jean-Baptiste, QC, Canada) and 1% penicillin–streptomycin (Wisent, Saint-Jean-Baptiste, QC, Canada).

### 2.2. Primary Neurons

Dissociated cortical neurons were prepared as previously described [22]. In brief, a cortical layer was dissected out of P0–P2 C57 and dissociated enzymatically (papain, 12 U/mL; Worthington, Lakewood, NJ, USA) and mechanically (trituration with flame-polished Pasteur pipette). After dissociation, the cells were washed, centrifuged, and plated on poly-d-lysine-coated glass coverslips at a density of 1.25–2.5 × 10^5^ cells/mL. Growth media consisted of Neurobasal and B27 (50:1), supplemented with penicillin–streptomycin (50 U/mL; 50 U/mL) and 0.5 mM L-glutamax (Thermo Fisher Scientific, Waltham, MA, USA). FBS (2%; Wisent, Saint-Jean-Baptiste, QC, Canada) was added at the time of plating. After 5 d, half of the media was changed without serum and with cytosine arabinoside (5 µM; Sigma-Aldrich St. Louis, MO, USA) to limit proliferation of non-neuronal cells. Twice a week thereon, half of the growth medium was replaced with serum- and cytosine arabinoside-free medium.

### 2.3. Construct Creation

The GFP-PTCHD1 in the pDEST-53 vector construct was used to generate the panel of PTCHD1 point mutants. All single-nucleotide substitution point mutations in the N-term GFP form were created with ligation-independent PCR cloning technique. Primers for the sense strand and anti-sense strand were designed for each independent mutation as seen in Appendix A Table A2. The new construct was created from a PCR reaction that amplified the entire plasmid harbouring the substitution using the high-fidelity polymerase Q5 according to the manufacturer’s instructions (New England Biolabs, Whitby, ON, Canada). The unpurified PCR product was then treated with DpnI to digest the methylated template DNA which was subsequently transformed into DH5α. Final constructs were verified through Sanger sequencing (ACGT Corporation, Toronto, ON, Canada). The SAP102 construct was kindly provided by Prof. Igor Stagljar (University of Toronto).

All PTCHD1 WT and point mutant constructs were inserted into the 3rd-generation lentivirus vector, pUltra (#24129; Addgene, Watertown, MA, USA), which includes a puromycin resistance gene. Using PCR amplification, a unique NheI site was generated upstream of the start codon of the PTCHD1 constructs with a blunt end after the stop codon. The PCR product was ligated into the NheI and HincII sites in the pUltra vector. All clones were verified with restriction enzyme diagnostic digest.

The mouse ortholog of *Ptchd1*, which exhibits 98.1% sequence conservation (871 of 888 amino acids) with human *PTCHD1*, was amplified using high-fidelity Q5 polymerase from cDNA that was generated from RNA obtained from P19-induced neural cells. PCR amplicons were digested and ligated into pcDNA3.1 myc-His B expression vectors (Thermo Fisher Scientific, Waltham, MA, USA), with a 3xFlag epitope tag also fused to the N-terminus of *Ptchd1*. Site-directed mutagenesis was subsequently used, as previously described, to generate the Pro32Arg, Pro75Gln, Lys181Thr, Gly303Arg, Phe549Cys, and ∆ITTV point mutants of Ptchd1. Final constructs were verified through Sanger sequencing (The Centre for Applied Genomics, Toronto, ON, Canada). Primer sequences for cloning and *Ptchd1* site-directed mutagenesis are provided in Appendix A Table A2(B).

### 2.4. Lentivirus Production and Transduction

HEK293T cells were seeded in a 6 cm plate. At ~80% confluency, transfection was performed with PEI (2 mg/mL) at a 2 µL:1 µg ratio of PEI to DNA in serum-free Dulbecco’s Modified Eagle Medium. Three plasmids were transfected: 1 µg of pLenti-PTCHD1, 0.75 µg psPAX2 packaging plasmid (#12260; Addgene, Watertown, MA, USA), and 0.25 pCMV-VSV-G envelope plasmid (#8454; Addgene, Watertown, MA, USA). After a 15 min incubation with PEI, the solution was added to the cells. Media were collected after 48 h and stored in 1 mL aliquots at −80 °C.

Primary neurons were used for lentiviral transduction at D3. Transduction of cells was performed at 1:5–1:2 of viral media to total culture media and incubated for 48 h.

### 2.5. Western Blotting and Co-Immunoprecipitations

All Western blots and co-immunoprecipitations were performed using lysates from transiently transfected HEK293T cells, unless stated otherwise. HEK293T cells were grown to 70–80% confluency in 100 mm plates and transfected using 2 mg/mL polyethylenimine (PEI; MilliporeSigma Canada Ltd., Oakville, ON, Canada) at a 2 µL:1 µg ratio of PEI to DNA. Cell lysates were taken 48–72 h after transfection. Cell lysates were prepared by washing cells twice with ice cold PBS, pH 7.4 (137 mM NaCl, 2 mL KCl, 10 mM Na_2_HPO_4_, 2 mM KH_2_PO_4_), and then adding 1% NP-40 lysis buffer (50 mM Tris pH 8.0, 120 mM NaCl, 1% NP-40) containing protease and phosphatase inhibitors (10 mM NaF, 1 mM PMSF, 2 µg/mL leupeptin, 2 µg/mL aprotinin, 1 mM sodium orthovanadate). For the Western analysis of protein expression, 50 µg of lysate was used, and samples in 4×-sample buffer were then incubated at 37 °C for at least 20 min prior to loading on the gel in order to avoid the boiling-induced aggregation of SSD-containing transmembrane proteins [13,23]. Blots were developed using Western Lightning PLUS ECL (PerkinElmer, Waltham, MA, USA) and imaged on a MicroChemi 2.0 Imager (FroggaBio, Concord, ON, Canada). Antibodies and concentrations used for Western blots can be seen in Appendix A Table A3.

Immunoprecipitation experiments were performed using 250 µg of total protein, made up to 500 µL total volume with 1% NP-40 buffer. Samples were incubated overnight at 4 °C with primary antibody. Immunocomplexes were bound to Protein G-Agarose (Thermo Fisher Scientific, Waltham, MA, USA) or Protein A-Agarose (Thermo Fisher Scientific, Waltham, MA, USA) beads and washed 5× with 1% NP-40 buffer, and blots were prepared as described above.

For co-immunoprecipitation experiments involving ^3xFlag^Ptchd1 and SAP102b^myc^, 250 µg of total protein samples were made up to 500 µL total volume with 1% NP-40 buffer and incubated overnight at 4 °C with 1 µL of mouse anti-Flag primary antibody (#F1804; MilliporeSigma Canada Ltd., Oakville, ON, Canada). Subsequently, samples were incubated with 50 µL of Protein G-conjugated Dynabeads (#10003D; Thermo Fisher Scientific, Waltham, MA, USA) for two hours at room temperature, and immune complexes were eluted (75 mM Glycine-HCl, pH 2.7) for five minutes at room temperature. For Western blot detection of ^3xFlag^Ptchd1 and SAP102b^myc^, rabbit anti-DYKDDDDK (#D6W5B; New England Biolabs, Whitby, ON, Canada) and rabbit anti-myc (#71D10; New England Biolabs, Whitby, ON, Canada) primary antibodies were used, respectively, followed by the anti-rabbit HRP-conjugated secondary antibody (#W4011; Promega, Madison, WI, USA).

### 2.6. Glycosylation Assay

*N*-linked glycosylation processing of PTCHD1 was determined using Endo-β-N-acetylglucosaminidase H (Endo H) and Peptide-N^4^-(N-acetyl-β-glucosaminyl)-asparagine amidase F (PNGase F) as we previously described [13]. Briefly, PTCHD1 mutants were transiently expressed in HEK293T cells. Cells lysed in 1% NP40 buffer and 50 µg of protein lysate were made up to a total volume of 20 µL containing ddH_2_O and the appropriate NEB enzyme buffers. The samples were treated with either (i) no enzyme, (ii) 500U Endo H (New England Biolabs, Whitby, ON, Canada), or (iii) 500U PNGase F (New England Biolabs, Whitby, ON, Canada) for 1 h at 37 °C. Proteins were resolved using SDS-PAGE, and the migration of the samples was determined via Western blot analysis.

### 2.7. Protein Stability Assay

To determine the stability of PTCHD1 mutant proteins, HEK293T cells transiently expressing these proteins were treated with 50 µg/mL of the protein synthesis inhibitor, cycloheximide (CHX), for the described lengths of time. Cell lysates were prepared as described previously, and the expression levels of PTCHD1 were determined via Western blot analysis. Relative expression of PTCHD1 protein was quantified through densitometry using ImageJ software (Version 1.51). PTCHD1 signal was normalized to β-tubulin. Independent experiments were performed at least 3 times per construct. Statistical analysis was performed on GraphPad Prism.

### 2.8. Co-Localization Image Acquisition and Analysis

For co-localization of PtchD1 mutants in HEK239 cells, sterile 13 mm coverslips (Sarstedt) were placed into 24-well culture plates (Falcon) and coated with poly-D-lysine solution (#1804; MilliporeSigma Canada Ltd., Oakville, ON, Canada). HEK293T cells were re-seeded at low density onto the coated coverslips. The following day, Lipofectamine 3000 (Thermo Fisher Scientific, Waltham, MA, USA) was used to co-transfect 10 ng of the *3xFlag-Ptchd1* expression plasmids and 490 ng of the inert plasmid pBV-Luc (addgene.org #16539). After 24 h, cells were rinsed with PBS and fixed with ice-cold 100% methanol at −20 °C for 15 min. Cells were then washed twice with PBS and incubated in blocking solution (10% goat serum (Cell Signaling; Danvers, MA, USA) in PBS supplemented with 0.1% Tween-20) for one hour at room temperature. Cells were incubated with primary antibodies overnight in a humidified chamber at 4 °C. After 15 h, cells were washed three times with PBS followed by incubation with Alexa Fluor-conjugated secondary antibodies for 1 h at room temperature. Cells were then washed three times with wash buffer, followed by incubation with NucBlue reagent (Thermo Fisher Scientific, Waltham, MA, USA). Coverslips were mounted on glass microscope slides using Dako mounting medium (Agilent; Santa Clara, CA, USA).

Images were acquired using a Leica TCS SP8 (Leica Microsystems, Wetzlar, Germany) confocal microscope and the Leica Application Suite X (LASX Version 1.4.5) software. Three lasers were used to acquire each image: 405 nm (66.6% intensity), 488 nm (5.2% intensity), and 552 nm (2.0% intensity). Consecutive z-stacks were acquired with a z-interval of 0.6 µm under 63× magnification. For each experimental condition, three biological replicates (each consisting of three consecutive z-stacks for 6–7 separate images) were analyzed by an independent technician who was blinded to each missense variant. Pixel intensity thresholds were identically adjusted for all images: (1) 3xFlag (minimum 30; maximum 255) and (2) Calnexin/At1a (minimum 15; maximum 230). To quantify overlapping intensity values of 3xFlag-Ptchd1 and Calnexin or At1a, the Pearson correlation coefficient (PCC) was calculated using the JACoP (Version 2.1.4) plugin in FIJI using default settings. To determine statistical significance, a one-way analysis of variance (ANOVA) was used, followed by a Tukey’s honestly significant difference (HSD) test to compare each missense variant with the wildtype.

Images from three independent co-staining experiments were analyzed, with 6–7 images acquired per co-staining. Data points from all technical replicates were plotted.

## 3. Results

### 3.1. Characterization of PTCHD1 Mutants

We identified PTCHD1 missense variants from clinical studies where variants were identified in male individuals presenting with a neuro-developmental disorder and were not present in the control database (gnomAD: gnomad.broadinstitute.org; from >182,000 exomes + genomes sequenced). For these variants, we used two methods: (i) the Condel missense prediction meta-algorithm, which combines predictions from five algorithms [24] and (ii) the Combined Annotation-Dependent Depletion (CADD) [25] to predict whether the substitutions are likely to be deleterious.

**Figure 1:**
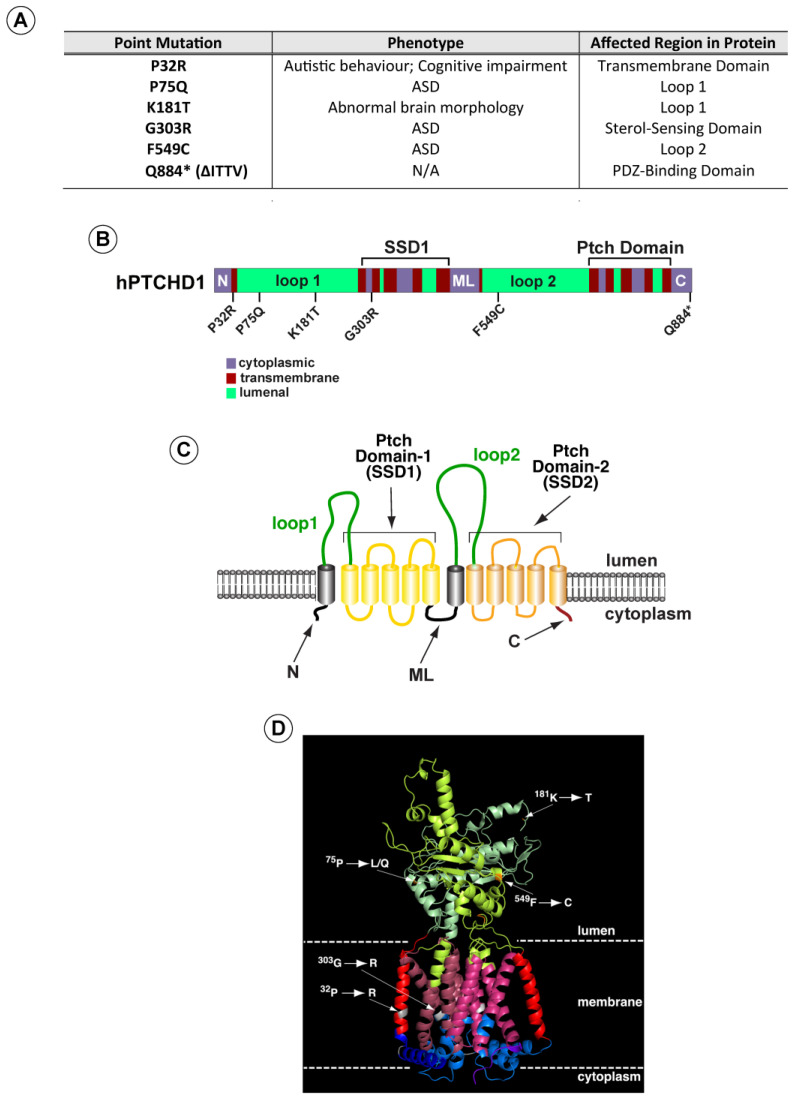
PTCHD1 graphics depicting point mutations and predicted structure. (**A**) Summary of point mutants in PTCHD1 from clinical studies (see also Appendix A Table A1). (**B**) Linear schematic of PTCHD1 indicating structural domains and locations of point mutations. (**C**) Cartoon of PTCHD1 illustrating the predicted topological orientation of specific regions in the membrane. (**D**) A predicted 3D structure of PTCHD1 based on the resolved cryo-EM structures of NPC1 and Ptch1. Locations of the point mutations in the hypothetical structure are indicated. Note that the cytoplasmic domains cannot be resolved for this class of proteins and are, therefore, absent.

Based on these analyses, we selected a subset of variants (Figure 1A) (i) predicted by both methods to be deleterious, (ii) spanning the PTCHD1 protein, and (iii) representing mutations in distinct structural regions. These regions included the first and second luminal domains, “Loop 1” and “Loop 2”, and the two transmembrane modules that produce the 3D structures resembling sterol sensing domains (“Ptch-domains”), SSD1 and SSD2 (Figure 1B–D). For comparison purposes, we also included a nonsense mutation (∆Ile-Thr-Thr-Val (∆ITTV)) that disrupts the predicted C-terminal PDZ-binding motif, although this deletion does not correspond with a known clinically reported mutation.

As the linear cartoon in Figure 1B illustrates, two of these mutations (Pro32Arg and Gly303Arg) produce amino acid changes in α-helical regions that are predicted to traverse the membrane. In contrast, Pro75Gln, Lys181Thr, and Phe549Cys alter amino acids in the luminal domains. Using Phyre^2^ to align the sequence for PTCHD1 with that of human NPC1 and Ptch1, whose 3D structures have been determined previously [10,19,20,21,26,27], we used PyMol (Version 2.5.0) to generate a hypothetical three-dimensional structure for PTCHD1 (Figure 1D). For all regions except the N, ML, and C cytoplasmic domains, this predicted model is nearly identical to a recent 3D model predicted using AlphaFold [28] (see Appendix A Table A1). As illustrated on the hypothetical model in Figure 1D, the Pro32Arg and Gly303Arg mutations are expected to disrupt helical structures in the first two highly conserved alpha-helices in the T-class of SSD-containing proteins [29]. G303R specifically affects the first helix in the SSD1-like domain, being that the integrity of this domain is essential for the activities of both NPC1 [30] and Ptch1 [31,32,33,34].

**Figure 2:**
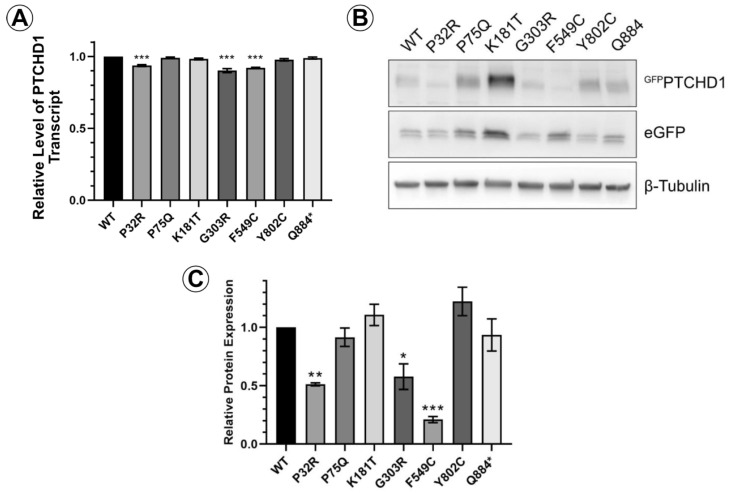
PTCHD1 mutants in stably expressed cells vary in protein expression levels. (**A**) Relative levels of stably expressed ^GFP^PTCHD1 transcript in bulk cultures of HEK293 cells. (**B**) Representative Western blot ^GFP^PTCHD1, eGFP and β-tubulin for stable lines expressing the PTCHD1 mutants. (**C**) Quantification of relative levels of expression of individual mutants stably expressed in HEK293 cells. Data displayed as mean ± SEM, *n* = 3 independent experiments. Data were analyzed using one-way ANOVA followed by Dunnett’s multiple comparisons test of means to the control (WT) using *t*-test. * *p* ≤ 0.05, ** *p* ≤ 0.01, *** *p* ≤ 0.001.

We first sought to determine differences in the relative levels of protein expression due to mutations on PTCHD1. An eGFP cassette was fused to the N-terminal end of PTCHD1 to create the ^GFP^PTCHD1 construct, which was then cloned in frame with the eGFP cassette through the P2A site in the pULTRA lentiviral vector. This arrangement produces a single transcript encoding an eGFP-P2A-^GFP^PTCHD1 fusion protein. The P2A site facilitates proteolytic cleavage between the eGFP and ^GFP^PTCHD1 proteins, allowing for a direct comparison of their expression. Figure 2A demonstrates that in HEK293 cells, the level of mRNA transcripts encoding the eGFP-P2A-^GFP^PTCHD1 protein for all mutants was essentially identical, differing by less than 10%. In contrast to these levels of mRNA transcript, Figure 2B illustrates that the levels of protein for these mutants varied considerably. The quantification of ^GFP^PTCHD1 protein expression was normalized to those of the eGFP alone and β-tubulin. These levels, relative to WT, can be seen in Figure 2C where several of the point mutations are expressed at considerably lower levels compared to the independently expressed eGFP. Thus, despite their essentially identical levels of mRNA expression, the resultant proteins show considerable heterogeneity in their level of protein expression.

### 3.2. Distinct Processing of PTCHD1 Mutants

**Figure 3:**
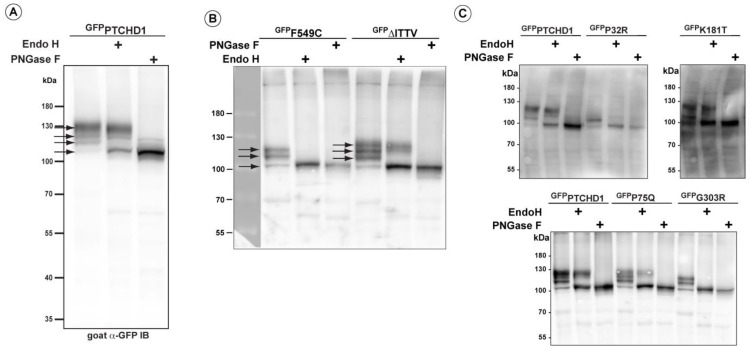
Altered post-translational processing of distinct PTCHD1 mutants. HEK293T cells were transfected with constructs expressing wild-type ^GFP^PTCHD1 or the point mutants, treated EndoH, or PNGase and resolved using SDS-PAGE on Western blots. (**A**) Wild-type ^GFP^PTCHD1 alone. (**B**) ^GFP^F549C and ^GFP^∆ITTV are processed differently, and only ^GFP^∆ITTV has the apparent normal processing. (**C**) Analysis of processing for ^GFP^P32R, ^GFP^K181T, ^GFP^P75Q, and ^GFP^G303R.

A close inspection of the protein bands seen in ^GFP^PTCHD1 in Figure 2B shows that these mutant proteins have distinct patterns of migration under denaturing conditions in SDS-PAGE gels. Given the variation in expression patterns and levels of these mutant proteins, we characterized their post-translational processing, protein stability, and subcellular localization. Figure 3A illustrates that wild-type ^GFP^PTCHD1 is processed to complex *N*-linked glycosylated forms. Here, the migration of the bulk of ^GFP^PTCHD1 is unaffected by treatment with EndoH, as a stronger band persists near the 130 kDa mark, indicating the processing of the *N*-linked glycosylated moieties to more mature, complex structures for the wild-type protein. Figure 3B illustrates mutants with contrasting patterns of processing. In the case of the mutant protein that deletes the PDZ-binding motif, ∆ITTV, a pattern of slower migrating species similar to the WT ^GFP^PTCHD1 is evident, the slowest migrating species (upper arrow) also being resistant to EndoH. In contrast, the Phe549Cys point mutant exhibits only two slower-migrating species, both of which are susceptible to EndoH, consistent with this protein not being processed through Golgi-dependent transport pathways that generate mature *N*-link glycosylated proteins. Using the same analysis (Figure 3C), the point mutants Pro75Gln and Lys181Thr both gave rise to slow-migrating, EndoH-resistant bands similar to WT ^GFP^PTCHD1. In contrast, Pro32Arg and Gly303Arg did not exhibit mature glycosylated forms since all forms are susceptible to EndoH activity. Thus, these different point mutants distinctly alter the apparent post-translational processing of PTCHD1.

**Figure 4:**
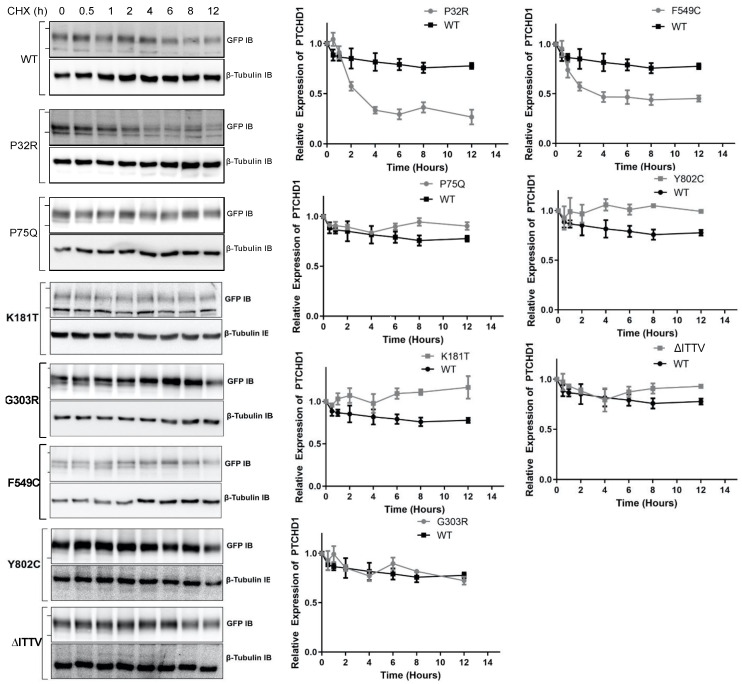
Point mutants alter the stability of the Pro32Arg and Phe549Cys variants. HEK293T cells were transfected with one of the ^GFP^PTCHD1 point mutants and treated with cycloheximide in 50 µg/mL for the time points indicated. *Left side*—Representative Western blots for time course of CHX-treated HEK293 cells transiently expressing WT ^GFP^PTCHD1, ^GFP^P32R, ^GFP^P75Q, ^GFP^K181T, ^GFP^G303R, ^GFP^F549C, ^GFP^Y802C, and ^GFP^∆ITTV mutants. *Right side*—Quantification of Westerns blots. Relative levels of mutants were compared to WT. Error bars represent standard error of the mean value of at least 3 independent experiments.

The fidelity of glycosylation plays a crucial role in stabilizing the protein expression in the cell. Given that specific point mutations modify the *N*-linked glycosylation of ^GFP^PTCHD1, the stability of these mutant proteins was measured to determine the lack of maturation of glycosylated mutants correlated with the altered protein half-life. As shown in Figure 4, the WT ^GFP^PTCHD1 protein exhibited a half-life beyond 12 h, determined by HEK293 cells transiently expressing ^GFP^PTCHD1 and treated with cycloheximide (CHX). Similarly, the point mutant, Pro75Gln, exhibited similar stability relative to WT ^GFP^PTCHD1, consistent with its apparent normal processing. In contrast, the protein half-life of Pro32Arg, which exhibited immature processing, was decreased to 2.5 h. Figure 4 also shows that the concordance between processing and protein half-life is evident for the mutants Lys181Thr and Phe549Cys, and the C-terminal truncation, ∆ITTV. Curiously, the Gly303Arg mutant, which also failed to be processed to more mature forms, had a half-life similar to the wild-type protein.

**Figure 5:**
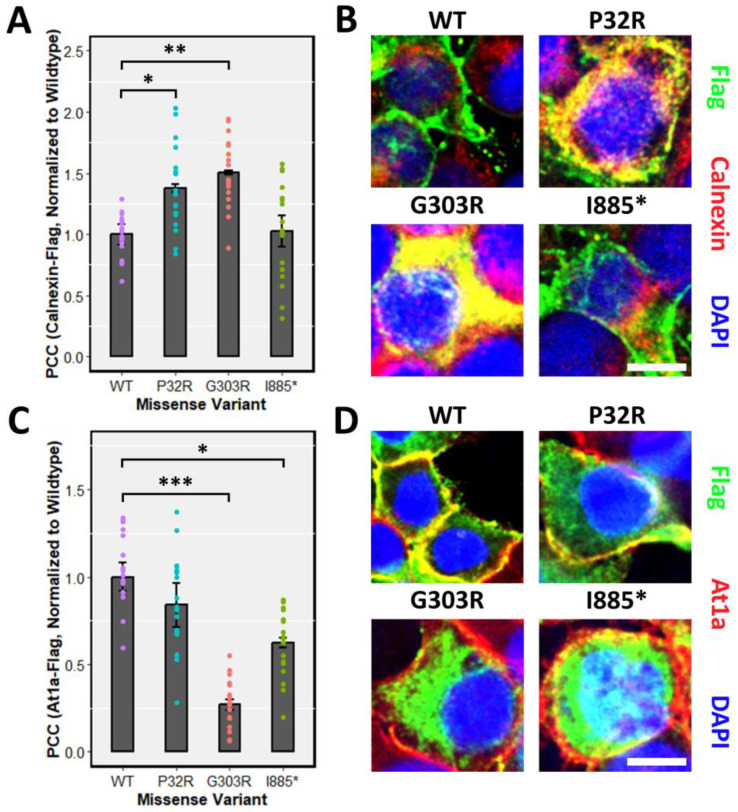
ER retention and impaired plasma membrane localization of point mutants. Immunofluorescence was performed, and co-localization was quantified using the PCC in HEK293T cells transfected with ^3xFlag^Ptchd1, ^3xFlag^Pro32Arg, ^3xFlag^Gly303Arg, or ^3xFlag^Ile885* and co-stained with either (**A**) an endogenous ER marker, Calnexin, or (**C**) an endogenous plasma membrane marker, At1a. Representative fluorescent images (Flag, green; Calnexin or At1a, red) for each missense variant are shown in (**B**,**D**). Scale bar represents 10 µm. Data are expressed as the mean ± SEM, and PCC values for each missense variant are normalized to wildtype Ptchd1. (* *p* < 0.05; ** *p* < 0.01; *** *p* < 0.001).

Proper post-translational modifications play a fundamental role in subcellular targeting. Due to the defects of processing ^GFP^PTCHD1 mutants harbouring specific point mutations, the subcellular localization may be affected. Specifically, proteins not susceptible to full EndoH cleavage may experience aggregation in the ER or Golgi, preventing their proper localization. We first tested the localization of the various mutants in transient assay in HEK293T cells. Consistent with the inability of ^GFP^Pro32Arg and ^GFP^Gly303Arg to be fully glycosylated, both ^3xFlag^Pro32Arg and ^3xFlag^Gly303Arg exhibited ER retention, as inferred by the increased co-localization with the ER marker Calnexin relative to WT ^3xFlag^PtchD1 (Figure 5A). Concordant with mature glycosylation evident in the GFP-tagged ΔITTV point mutant, ^3xFlag^Ile885* demonstrated a similar degree of co-localization with Calnexin as WT ^3xFlag^PtchD1. As shown in Figure 5C, the defect in the processing of ^3xFlag^Gly303Arg is associated with impaired plasma membrane trafficking, as indicated by attenuated co-localization with the plasma membrane marker At1a relative to WT ^3xFlag^PtchD1. Despite the apparent normal processing of ^3xFlag^Ile885*, decreased plasma membrane trafficking was also observed in this point mutant. Finally, despite ^3xFlag^Pro32Arg displaying ER retention, a statistical difference in plasma membrane localization was not observed in this point mutant relative to WT ^3xFlag^PtchD1.

**Figure 6:**
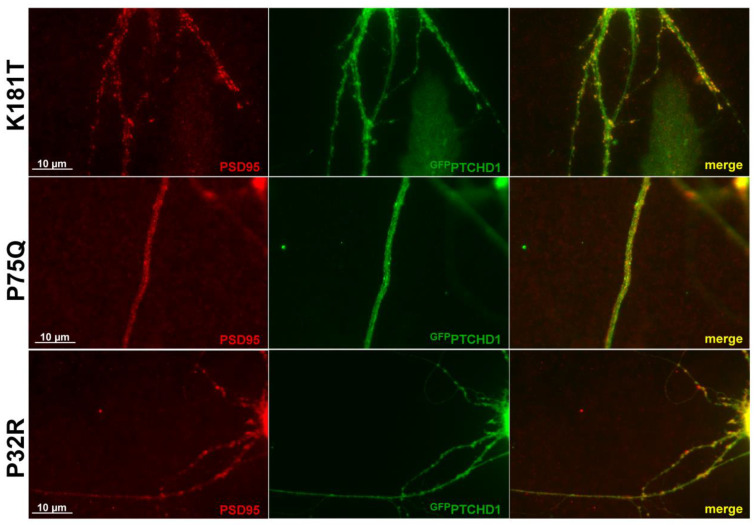
PTCHD1 constructs localize to endogenous PSD95 in primary neurons. Immunofluorescence was performed in primary neurons transiently expressing ^GFP^PTCHD1 mutants, ^GFP^Pro32Arg, ^GFP^Pro75Gln, or ^GFP^Lys181Thr (green) co-localized with endogenous PSD95 (Red).

Previous studies showed that ^GFP^PTCHD1 localizes with PSD95 in neuronal processes [17]. As Figure 6 shows for primary neurons, the lentiviral-mediated expression of the Pro75Gln, Lys181Thr, and Pro32Arg mutants showed that they all retained their ability to co-localize with PSD95 in neuronal processes. Although Pro32Arg showed a statistically significant amount of ER retention, Figure 6 indicates that there is a small population that can successfully localize to PSD95. Thus, regardless of their processing and stability, these mutants do not exhibit altered intracellular localization.

**Figure 7:**
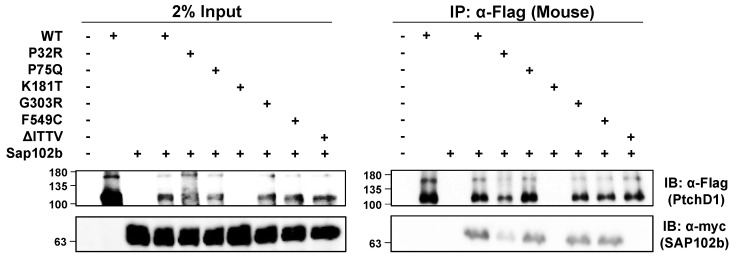
PTCHD1 interacts and co-localizes with SAP102b. *Left Side*—HEK293T cells were transiently transfected with either ^3xFlag^Ptchd1 or one of the 3xFlag-tagged point mutants (P32R, P75Q, G303R, F549C, or ΔITTV) and SAP102b^myc^ and resolved using SDS-PAGE on Western blots. *Right Side*—Co-immunoprecipitation of SAP102b^myc^ with wildtype ^3xFlag^Ptchd1 or Ptchd1 point mutants. Each IP was divided in half, run on two gels, and probed with either anti-flag (Ptchd1; upper panel) or anti-myc (SAP102b; lower panel).

**Figure A1:**
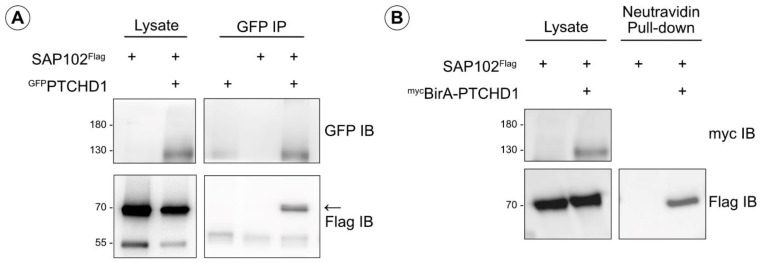
SAP102 as a potential interactor of PTCHD1. (**A**) Co-immunoprecipitation of Flag-tagged SAP102 (70 kDa) with GFP-tagged PTCHD1 (130 kDa). Transiently expressed in HEK293T cells, GFPPTCHD1 was immunoprecipitated, and SAP102Flag was detected using mouse α-Flag antibody. (**B**) BioID assay detecting biotinylation of SAP102Flag from mycBirA-PTCHD1. A pull-down with Neutravidin agarose beads isolated biotinylated proteins, and SAP102Flag was detected in this sample.

The isolated C-terminal domain of PTCHD1 was shown previously to bind to the PDZ-containing, postsynaptic scaffolding proteins, PSD95 and SAP102 [17]. Using ^myc^SAP102b, an isoform that has deleted the first two PDZ domains [35,36], and the mouse ortholog of ^3xFlag^Ptchd1, Figure 7 shows that all of the WT ^3xFlag^Ptchd1, as well as the Pro75Gln, Gly303Arg, Phe549Cys, and Pro32Arg mutants co-immunoprecipitated ^myc^SAP102b. The newly derived Flag-tagged Lys181Thr mutant failed to express for an unknown reason, and no statement can be made regarding its ability to co-immunoprecipitate ^myc^SAP102b. Appendix A Figure A1 illustrates that the GFP-tagged version of PtchD1 interacts with SAP102b identically to the Flag-tagged version of PtchD1. That this interaction was mediated by the PDZ-binding motif at the very end of the C-terminus of Ptchd1 was confirmed using the deletion mutant, ∆ITTV, which harbours a deletion of the PDZ-binding motif.

## 4. Discussion

There exists a high density of synaptic scaffolding proteins at the PSD that organize neurotransmitter receptors, in part, by utilizing their PDZ domains to bind to cellular elements. Previously, interactions between the C-terminal PDZ binding motif in PTCHD1 with synaptic scaffolding proteins, PSD95 and SAP102, were described [16,17]. However, neither of these previous studies verified the interaction with the full-length PTCHD1 protein. We showed the interaction between full length PTCHD1 and SAP102. Further, using the shorter construct of SAP102b isoform, we demonstrated that the third PDZ domain of SAP102 was sufficient for PTCHD1 binding. SAP102 links NMDARs to excitatory type 1 synapses and mediates AMPAR-regulatory activities [35,37,38]. This highly mobile member of the MAGUK family is not sequestered at the PSD as it has been shown to interact with NMDAR subunits in the secretory pathway, specifically the ER [36,39]. SAP102 also mediates NMDAR exocytosis, an activity distinct from those mediated by PSD95 [40]. Truncating mutations of *DLG3*, the gene encoding SAP102, have been associated with X-linked retardation [41,42] and XLID with substantial impairment in cognitive abilities and social and behavioural adaptive skills [43]. Therefore, SAP102 has been proposed to be a plausible candidate gene for ASD [44]. Its interaction with PTCHD1 may implicate that the mechanism employed for PSD-targeted trafficking and endocytosis of PTCHD1 is mediated by SAP102. In this vein, we have shown that all Ptchd1 point mutants studied here, except for ∆ITTV which lacks the four-amino acid (Ile-Thr-Thr-Val) putative PDZ-binding domain, possess the ability to bind to SAP102b. These data suggest that the neurodevelopmental consequences of these specific *PTCHD1* mutations may arise independently of its interaction with SAP102 within dendritic spines.

Advances in genome sequencing and genetic analyses have identified an increasing number of genes responsible for ASD by detecting de novo mutations linked to ASD. These mutations can be CNVs or single-base-pair mutations; however, missense mutations are less informative because their impact on the protein is unknown. ASD-linked single missense mutations have been described in proteins involved in the dendritic spines such as *SHANK3, NLGN4*, and *ACTN4* [45,46,47,48]. Single missense mutations have also been identified in *PTCHD1* from individuals with ASD or ASD-associated disorders. In this study, we analyzed a number of clinically relevant PTCHD1 point mutations and found that Pro32Arg, Gly303Arg, and Phe549Cys have the most adverse effects on the protein. These mutations affect *N*-linked glycosylation in post-translational modifications, with some resulting in protein destabilization. However, these mutants were not observed to cause aggregation in the ER.

The deleterious consequences of processing and stability that arise from the Pro32Arg and Gly303Arg mutations, respectively, are of particular interest. While the specific activities of PTCHD1 in dendritic spines remains unresolved, the primary and 3D structures of PTCHD1 suggest that it may harbor cholesterol transport activities similar to the related proteins, NPC1 [11,12,49,50,51] and Ptch1. Interestingly, both Pro32Arg and Gly303Arg in PTCHD1 are in similar positions to the residues in Ptch1 that were recently shown to be involved in an apparent complex with a cholesterol moiety observed in the cryo-EM of Ptch1 [21,27]. Located on the α-helices, TM1 and TM3, respectively, the sidechains of these residues are involved in coordinating the orientation of a likely cholesterol molecule in this region. Given the fundamental roles of cholesterol in the localization and activities of the synaptic transmitters [52,53,54,55,56], we suggest that these mutations in PTCHD1 may specifically alter synaptic signaling due to the impaired localized transport of cholesterol in dendritic spines.

When observing the extent of maturation of *N*-linked glycosylation in PTCHD1 mutants, we hypothesized that the inherent stability of PTCHD1 could be altered due to an inability to be properly glycosylated. Proteins with post-translational modifications that are not properly matured do not typically proceed through the ER and Golgi and are subjected to early degradation. Our protein stability time course assay showed only two mutants, Pro32Arg and Phe549Cys, with a shorter half-life than WT. Both mutants exhibited some processing, these forms being susceptible to cleavage by Endo H indicating that they were not processed to the more mature Endo H-resistant forms. These results illustrate that single amino acid substitutions may result in decreased PTCHD1 protein stability as immature stages of *N*-linked glycosylation may not achieve proper folding of the protein and may lead to early degradation of the protein. The significance of this decreased stability requires further analysis.

In conventional protein-processing pathways, alterations in glycosylation may result in aggregation in the ER, causing ER stress and preventing the trafficking to subcellular locations. Correspondingly, immunofluorescence staining showed that the mutants Pro32Arg and Gly303Arg displayed overlapping aggregation with the co-stain of an ER marker, Calnexin. Consistent with this presumptive ER retention, the mutant Gly303Arg also demonstrated reduced overlap with the co-stain of the plasma membrane marker, At1a. The mutant with deletion of the PDZ-binding domain, ∆ITTV, did not exhibit ER retention, but did show impaired membrane localization, consistent with the role of PDZ domains in localizing their respective ligands to the correct plasma membrane domain [57]. Indeed, the staining of PTCHD1 mutants was seen throughout the cell with no observable differences in localization between the WT PTCHD1 protein and the mutants. Likewise, regardless of whether mutants were fully processed, they were observed to co-localize with PSD95 in neuronal processes. We propose that these mutants may represent hypomorphic or null variants whose principal activities in the PSD are crippled or lost, respectively, despite their ability to localize to the PSD95-containing dendritic structures. While potentially mediating localized fluxes of cholesterol seems possible, analogous to the activities of the structurally related proteins, NPC-1 and Ptch1, the uncharacterized activities of PTCHD1 remain speculative.

## 5. Conclusions

Taken together, our data suggest that the PTCHD1 missense mutants under investigation may exert an etiopathogenic effect through the reduced lifespan of the protein and, thus, reduced bioavailability, rather than through the disruption of the transport of PTCHD1 to its functional destination.

## Data Availability

The Phyre^2^ alignment of the PTCHD1 sequence with NPC1 and PTCH1 sequences, as well as the predicted PTCHD1 structure generated by PyMol, can be made available upon request.

## Abbreviations

ASD, Autism spectrum disorder; CHX, cycloheximide; PEI, polyethylenimine; RA, retinoic acid; RND, resistance nodulation division; SSD, sterol-sensing domain; TM, transmembrane; XLID, X-linked intellectual disability.

## Appendix A

**Table A1:** Selected PtchD1 variants for functional analysis.

Mutation	Genomic (hg19); cDNA (NM_173495.2) Coordinates	Source, Subject ID	Inheritance	Minor Allele Frequency (gnomAD)/# Hemizygotes	Prediction: Condel; CADD PHRED-like Scaled C-Score	Reported Phenotype
Pro32Arg	ChrX:23353087; c.95C>G	DECIPHER: 284363	Mat	0/0	Deleterious; 26.8	Autistic behavior; cognitive impairment
Pro75Gln	ChrX:23353216; c.224C>A	MSSNG: AU3794302	Mat	0/0	Deleterious; 27.6	Autism spectrum disorder
Lys181Thr	ChrX:23397898; c.542A>C	Clinvar	nr	0/0	Deleterious; 21.9	Abnormality of brain morphology
Gly303Arg	ChrX:23398263; c.907G>A	ClinVar ID 417957	nr	0/0	Deleterious; 27.7	Autism
Phe549Cys	ChrX:23411281; c.1646T>G	Ptchd1-base.com	nr	0/0	Deleterious; 28	Autism spectrum disorder
∆ITTV	ChrX:23412285; c.2650C>T	No subject	na	0/0	38	na

nr = not reported. na = not applicable.

**Table A2:** (**A**) Primers used to generate PTCHD1 point mutations. (**B**) Primers used to for mouse *Ptchd1* cloning and site-directed mutagenesis.

Point Mutation	Sequence
(**A**)	
P32R Forward	5′-CACCCTGTCTTCTTCGCCTCGGCGCGGGTGCTCATCTCCATCCTGCTG-3′
P32R Reverse	5′-CAGCAGGATGGAGATGAGCACCCGCGCCGAGGCGAAGAAGACAGGGTG-3′
P75Q Forward	5′-GTTAACAGCCTCTTCCAGGTCAACCGCTCCAAGCACCGTCTCTACTCG-3′
P75Q Reverse	5′-CGAGTAGAGACGGTGCTTGGAGCGGTTGACCTGGAAGAGGCTGTTAAC-3′
K181T Forward	5′-ACATACCCAATCACTCACTTAACGGACGGGAGGGCTGTGTACAATGGG-3′
K181T Reverse	5′-CCCATTGTACACAGCCCTCCCGTCCGTTAAGTGAGTGATTGGGTATGT-3′
G303R Forward	5′-AAACCCTGGCTAGGCCTGCTCCGATTGGTGACCATAAGCCTGGCC-3′
G303R Reverse	5′-GGCCAGGCTTATGGTCACCAATCGGAGCAGGCCTAGCCAGGGTTT-3′
F549C Forward	5′-ACTACTGCCCAGCAAAAGTACTGCAGCAACTACAGTCCTGTGATT-3′
F549C Reverse	5′-AATCACAGGACTGTAGTTGCTGCAGTACTTTTGCTGGGCAGTAGT-3′
∆ITTV Forward	5′-ATATCGATAGTACCCGTGTGGTTGACTAAATTACAACAGTGTGA-3′
∆ITTV Reverse	5′-TCACACTGTTGTAATTTAGTCAACCACACGGGTACTATCGATAT-3′
(**B**)	
P32R Forward	5′-AGAGTGCTCATCTCCATCCTGCTC-3′
P32R Reverse	5′-CGCCGAAGCAAAGAAGACCG-3′
P75Q Forward	5′-CAAGTCAACCGCTCCAAGCACC-3′
P75Q Reverse	5′-GAAGAGGCTGTTGACTAGGTTGCG-3′
K181T Forward	5′-CAGATGGAAGGGCTGTGTATAATGGG-3′
K181T Reverse	5′-TTAAGTGAGTGATCGGATATGTGATAGCAAAATTGG-3′
G303R Forward	5′-CGGTTGGTGACCATAAGCCTAGC-3′
G303R Reverse	5′-AAGTAGGCCTAACCAGGGTTTGC-3′
F549C Forward	5′-GTAACAACTACAGTCCTGTTATTGGGTTTTAC-3′
F549C Reverse	5′-AGTACTTTTGGTGGGCAGTAGTG-3′
∆ITTV Forward	5′-TGAATAGGAGTTTAAACCCGCTGATCAGCCTC-3′
∆ITTV Reverse	5′-TTGGTCAACCACTCGAGTACTATCAATATCTACC-3′
Ptchd1 Forward (HindIII)	5′-CGTACG**AAGCTT**ATGCTGCGGCAGGTTCTG-3′
Ptchd1 Reverse (AgeI)	5′-GTACG**ACCGGT**TCACACTGTGGTTATTTGGTCAACCAC-3′

**Table A3:** Antibodies used for Western blots and immunofluorescence staining.

	Antibody	Source	Dilution
1° Antibody	Mouse α-Flag-Tag	Applied Biological Materials Inc. (Richmond, BC, Canada) Cat # G191	1:1000 WB IB
	Mouse α-Myc-Tag (9B11)	Cell Signaling Technologies (Danvers, MA, USA) #2276S	1:1000 WB IB; 1:400 IF
	Rabbit α-GFP-Tag	Invitrogen (Waltham, MA, USA) Cat #A11122	1:100 WB IB; 1:400 IF
	Goat α-GFP-Tag	Applied Biological Materials Inc. (Richmond, BC, Canada) Cat # G096	1:1000 WB IB; 1:400 IF
	Goat α-Biotin	Cedarlane. (Burlington, ON, Canada) CLAS10-666	1:1000 WB IB
	Rabbit α-PSD95	Abcam (Waltham, MA, USA) ab18258	1:400 IF
	Mouse α-β-Tubulin	Applied Biological Materials Inc. (Richmond, BC, Canada) Cat # G098	1:5000 WB IB
	Mouse α-GM130	BD Transduction Laboratories (Mississauga, ON, Canada) Cat # 51-9001978	1:400 IF
HRP-Linked 2° Antibody	Donkey α-goat IgG	Santa Cruz Biotechnologies Inc.(Dallas, TX, USA) sc-2020	1:5000
	Horse α-mouse IgG	Cell Signaling Technologies (Danvers, MA, USA) #7076	1:5000
	Goat α-rabbit IgG	Cell Signaling Technologies (Danvers, MA, USA) #7074	1:5000
Immunofluorescence 2° Antibody	Donkey α-rabbit IgG Alexa Fluor 488	Invitrogen (Waltham, MA, USA) A21206	1:400 IF
	Donkey α-mouse IgG Alexa Fluor 568	Invitrogen (Waltham, MA, USA) A10037	1:400 IF
	Donkey α-rabbit IgG Alexa Fluor 568	Invitrogen (Waltham, MA, USA) A10042	1:400
	Donkey α-goat FITC	Jackson ImmunoResearch (West Grove, PA, USA) 705-095-003	1:400
	Phalloidin Alexa Fluor 350	Invitrogen (Waltham, MA, USA) A22281	1:20

**Table A4:** Single-cell RNAseq co-expression data extracted from adolescent mouse study (mousebrain.org; accessed on 3 September 2021 [58]), showing cell types with the highest expression of *Ptchd1* (top 20, highest to lowest), with expression values (log_2_(x + 1) transformed average molecule counts) for putative interactors, and for housekeeping genes.

			Study Gene	Putative Protein Interactors			Housekeeping Genes			
Index	Name	Description	*Ptchd1*	*Snapin*	*Dlg3*	*Dlg4*	*Gapdh*	*Actb*	*Sdha*	*Rpl37*
70	HBCHO3	Afferent nuclei of cranial nerves VI–XII	0.86	1.4	0.346	2.24	2.34	23.2	4.68	28.3
81	HBSER5	Serotonergic neurons, hindbrain	0.515	0.908	0.452	2.76	1.92	10.2	3.3	41
167	HBGLU8	Excitatory neurons, hindbrain	0.363	0.546	1.45	2.36	7.09	17.5	8.82	16.3
187	ENT6	Cholinergic enteric neurons	0.313	1.08	0.0679	1.5	8.09	9.63	1.1	20.8
79	HBSER2	Serotonergic neurons, hindbrain	0.266	0.836	0.384	1.86	1.72	10.9	3.39	27.9
82	HBSER4	Serotonergic neurons, hindbrain	0.25	0.668	0.415	2.15	1.23	7.84	2.37	30
165	HBGLU6	Excitatory neurons, hindbrain	0.246	0.521	0.652	1.1	3.36	8.55	5.66	11
183	ENT2	Nitrergic enteric neurons	0.224	1.61	0.149	1.04	11.9	20.1	1.57	24.1
189	ENT8	Cholinergic enteric neurons, VGLUT2	0.216	1.67	0.0307	1.9	14.5	16.8	1.53	38.7
182	ENT1	Nitrergic enteric neurons	0.214	1.29	0.213	1.21	13.3	14.6	2.29	21.4
68	DECHO1	Cholinergic neurons, septal nucleus, Meissnert and diagonal band	0.202	1.57	0.498	1.75	4.4	10.3	3.16	33.1
166	HBGLU7	Excitatory neurons, hindbrain	0.2	0.445	0.707	1.14	2.59	8.24	3.94	8.76
197	SYCHO1	Cholinergic neurons, sympathetic	0.2	3.07	0.266	1.07	14.1	5.53	3.13	23.8
80	HBSER3	Serotonergic neurons, hindbrain	0.195	0.839	0.206	2.24	1.5	5.65	1.74	30.8
162	HBCHO2	Cholinergic neurons, hindbrain	0.182	0.546	0.317	1.64	3.36	13	5.41	14.8
159	HBINH6	Inhibitory neurons, hindbrain	0.177	0.485	0.434	1.03	1.87	4.63	3.03	10.6
190	ENT9	Cholinergic enteric neurons	0.177	1.27	0.0238	2.05	9.82	26	1.51	30.5
120	HBGLU2	Excitatory neurons, hindbrain	0.165	0.421	0.244	0.864	1.3	5.82	2.21	13.7
115	SCGLU8	Excitatory neurons, spinal cord	0.133	0.2	0.0667	0.533	0.598	2.27	0.801	4.6
150	HBINH5	Inhibitory neurons, hindbrain	0.133	0.335	0.326	1.02	1.86	5.67	3.35	9.73
201	PSPEP5	Peptidergic (PEP1.2), DRG	0.133	1.97	0.0331	0.234	1.53	2.6	1.43	6.47
